# Early Onset Pancreatic Adenocarcinoma (EOPAC): presentation, clinical course and treatment outcomes in comparison to Average Onset Pancreatic Adenocarcinoma (AOPAC): a retrospective cohort study

**DOI:** 10.1186/s12885-024-12955-7

**Published:** 2024-10-18

**Authors:** Noha Rashad, Abdelrahman Gouda, Esraa Sabra, Mohamed A. Youssef, Hossam Alshazly, Sandra Samir

**Affiliations:** 1 Medical Oncology Department, Shefaa Al-Orman Oncology Hospital, Luxor, Egypt; 2 Department OF clinical oncology, Faculty of medicine, Suez University, PO Box 43221, Suez, Egypt; 3 Clinical oncology department, Aswan University, Aswan, Egypt; 4 Department of internal medicine, Medical branch, University of Texas medical branch, USA Galveston,; 5 Clinical pharmacy Department, Shefaa Al-Orman Oncology Hospital, Luxor, Egypt

**Keywords:** Early onset cancer, Pancreatic adenocarcinoma, Risk factors, Presentation, Clinic-pathological features, Therapy, Prognosis.

## Abstract

**Background:**

Pancreatic adenocarcinoma (PAC) is a disease of decimal prognosis, with around 50% of patients presenting with metastatic disease. Previous trials reported a high incidence of early onset pancreatic cancer (EOPAC) in Egypt, presenting about 25% of patients with PAC. The clinic-pathological features and prognosis of EOPAC needs more study.

**Patients and methods:**

A retrospective analysis of patients’ records at Shefa Al-Orman comprehensive cancer center database. Patients with histo-pathologically confirmed diagnosis of PAC. We categorized patients according to the age at diagnosis into EOPAC (≤ 50 years) and average onset PAC (AOPAC). Data on risk factors, family history, presenting symptoms, clinic-pathological features, treatment, and prognosis were extracted. Patients with histopathologically confirmed diagnosis of pancreatic cancer diagnosed between December 2016-December 2022 were included.

**Results:**

The study cohort consisted of 412 patients. EOPAC represented 20.3% of patients, with no significant differences in risk factors and family history compared to AOPAC. Duration of symptoms before diagnosis is longer in EOPAC, with the majority of EOPAC presenting with localized disease (23.8%) and locally advanced tumors (28.5%) compared to AOPAC. AOPAC presented more with metastatic disease (64% vs. 45.2%, *p* = 0.003). EOPAC are usually submitted to more aggressive treatment including radical surgery, neoadjuvant therapy, and aggressive chemotherapy regimens in metastatic disease. Disease free survival (DFS) of EOPAC was shorter than AOPAC (11 months vs. 17 months, *p* = 0.889), but overall survival OS was significantly longer in EOPAC (10 months vs. 6 months, *p* = 0.013).

**Conclusion:**

Patients with EOPAC in Egypt represent around 25% of cases. EOPAC tend to have a shorter disease free survival (DFS) in patients presenting with localized disease. The overall survival (OS) is longer in EOPAC compared to AOPAC. Further studies are mandatory to identify the epidemiological and risk factors of EOPAC in Egypt.

## Background

Pancreatic adenocarcinoma (PAC) arising from exocrine pancreatic cells is the seventh leading cause of cancer related mortality worldwide [1]. More than 50% of patients present with metastatic disease, 30–35% present with locally advanced disease and 10–15% present with localized resectable tumors [2]. Despite the availability of new chemotherapeutic regimens that improved survival in adjuvant and metastatic disease [3], the age-standardized 5 year survival rate ranges between 5 and 15% [4]. 

Between 1990 and 2019, the incidence of cancer diagnosis among young adults have increased by 79% and mortality have increased by 27.7%,^21^ the increase is more noted in high and high-middle sociodemographic index countries with the main increase noted in patients with digestive system cancers [5]. 

Pancreatic cancer incidence and mortality is rising in young individuals worldwide [6]. The definition of early onset pancreatic cancer (EOPAC) varies among studies, usually defined as ≤ 50 years at diagnosis [7]. EOPAC usually present with advanced stage disease, more aggressive tumors and tend to have a shorter OS compared to AOPAC [8]. In most registries, EOPAC represent around 8–10% of patients with PAC [9]. 

Smoking, diabetes, obesity and alcohol intake are known risk factors for early onset pancreatic adenocarcinoma patients (EOPAC) as well as average onset PAC patients (AOPAC) [10]. Patients with familial pancreatic cancer and hereditary cancer syndromes tend to be diagnosed at a young age [11]. However, patients with known predisposing genetic factors represent around 10% of EOPAC with the main increase seen in patients with sporadic pancreatic cancers [12, 13]. 

In Egypt, previous epidemiological studies showed unusually high incidence of EOPAC and higher mortality when compared to USA pancreatic cancer related mortality rates [14, 15]. Around 25% of cases were diagnosed in patients below 50 years old with wide variability in geographical distribution of cases among urban and rural areas [16]. Dietary factors were studied as a possible etiological factor responsible for the high EOPAC rates but with no conclusive findings [17]. 

We conducted this study aiming to describe EOPAC patient demographics, possible underlying risk factors, clinico-pathological features, treatment patterns and survival compared to AOPAC. And to summarize the possible differences between EOPAC and AOPAC and highlighting therapeutic challenges and research needs in this area.

## Patients and methods

### Setting

A retrospective analysis of patients’ records at Shefa Al-Orman comprehensive cancer center database. Patients with histopathologically confirmed diagnosis of pancreatic cancer diagnosed between December 2016-December 2022 were included.

### Patients

All patients diagnosed with pancreatic adenocarcinoma during the above-mentioned time interval were included, patients with neuroendocrine tumors, pancreatic intraepithelial neoplasms, and non-malignant neoplastic tumors were excluded.

Data on patient demographics, possible etiological and risk factors, presenting symptoms, clinical stage at diagnosis, therapeutic interventions and treatment outcomes were extracted from medical records. Revision of histopathological report for histopathological data verification, whether a biopsy or surgical specimens was carried out. Time of diagnosis and other survival measures were calculated using date of biopsy or surgical intervention as a starting point. Patients were classified as localized initially resectable, locally advanced borderline resectable and metastatic disease based on the local policies defining criteria of resectability adopted by multidisciplinary team.

Patients were categorized into early onset group EOPAC (defined as patients under the age of ≤ 50) and average onset group (AOPAC) for patients > 50 years old.

The study was conducted after hospital ethical committee approval and independent review board at Shefaa Al Orman cancer center (SOH-IRB). SOH-IRB registered at the office for human research protections, US department of health& human services and operates under federal wide assurance number FWA 00025406 and its registration number in the Egyptian ministry of health and population is RHDIRB 2,017,061,502.

### Statistical analysis

The SPSS (Statistical Package version 28) was used to analyze the data. The mean ± standard deviation or median (range) will be used to describe quantitative data. Frequency and percentage were used to summarize qualitative data. While qualitative data were described as Frequencies and percentages. Comparison between two groups for numerical variables was done using either the student t-test or Mann-Whitney –U test (non-parametric t-test).

From the date of diagnosis until death or the last follow-up date, the overall survival will be determined. Disease-free survivals were calculated from surgery date till the date of documented recurrence, metastasis, death or last follow up.

The Kaplan-Meier technique was used to conduct the survival analysis. The log-rank test was used to compare two survival curves. The Cox regression model was used in multivariate analysis to assess independent indicators for overall survival, risk assessment was computing using the hazard ratio and its 95% confidence interval. A p-value ≤ 0.05 will be considered significant.

## Results

### Patients

Four hundred thirty-three patients were identified as pancreatic cancer patients, presented to SOH between December 2016 till December 2022. After excluding patients with neuroendocrine tumors (Pan-NET), Patients with no histopathology confirmed diagnosis and non-malignant lesions, the final study cohort consisted of 412 patients. Of these, 84 patients were ≤ 50 years old (EOPAC) constituting 20.3% of the total study population and 328 patients were above 50 years old at diagnosis (AOPAC) constituting 79.7% of the patients. The median duration of follow-up was 6 months (range 1–70 months).

The mean age at diagnosis for the whole group was 60 years, the mean age at diagnosis for EOPAC was 43.6 (range 21–50) years and 64.7 (range 51–96) years for AOPAC. No difference in gender distribution was noted between EOPAC and AOPAC as shown in Table 1.

**Table 1:** Patient demographics, risk factors and tumor characteristics for all study participants, EOPAC compared to average onset PAC

	EOPAC (*n* = 84)	Average onset PAC(*n* = 328)	*P*
Age (mean, SD)y.	43.6 ± 6.6	64.7 ± 8.2	
**Sex**			
Male	51 (60.7%)	204 (62.2%)	
Female	33 (39.3%)	124 (37.8%)	0.803
**Performance status at presentation.**			
ECOG PS ≤1	70 (83.3%)	202 (61.5%)	
ECOG PS >1	14 (16.7%)	126 (38.6%)	0.001
**BMI mean ± SD**	23.6 ± 5.1	23.2 ± 4.9	0.247
**Comorbidity**			
Yes	27 (32.1%)	168 (51.2%)	0.01
No	27 (32.1%)	62 (18.9%)	
Missing	30 (35.8%)	98 (29.8%)	
**Type of comorbidity**			
Diabetes.	20 (23.8%)	135 (41.1%)	0.83
Hypertension.	6 (7.1%)	29 (34.5%)	
Cardiac disease.	1 (1.1%)	4 (1.2%)	
**Family history of malignancy.**			
No family history of cancer.	64 (76.19%)	257 (78.3%)	0.81
1st degree relative with PAC.	1 (1.2%)	5 (1.5%)	
1st degree relative with any type of cancer.	9 (10.7%)	28 (8.5%)	
Missing.	10 (11.9%)	38 (11.5%).	
**Weight and BMI**			
Weight mean ± SD	66.2 ± 14.7	62.1 ± 14.1	0.021
Height mean ± SD	167.5 ± 8.7	163.8 ± 9.3	0.001
BMI mean ± SD	23.6 ± 5.1	23.2 ± 4.9	0.247
**BMI classification**			
Underweight	18 (21.4%)	80 (24.3%)	
Normal	39 (46.4%)	157 (47.8%)	
Overweight	27 (32.2%)	91 (27.7%)	0.821
**Smoking**			
Yes	27 (32.1%)	91(27.7%)	
No	57 (67.9%)	237 (72.3%)	0.780
**Alcohol intake**			
No	24 (28.5%)	75 (22.8%)	
Missing	60 (71.5%)	253 (77.2%)	0.87
**Duration of symptoms before diagnosis (months)** Median (range)	2 (0.5–12)	1 (0.3–12)	0.030
**Presenting symptoms.**			
Jaundice	31 (36.9%)	110 (33.6%)	0.67
Weight loss	8 (9.5%)	17 (5.2%)	
Abdominal pain	39 (46.5%)	164 (50%)	
Missing	6 (7.1%)	37 (11.2%)	

### Risk factors

Data on comorbidities were missing for 128 patients (31%), AOPAC with known comorbidities were significantly higher than EOPAC (51.2% VS 32.1% respectively, *p* = 0.01). History of diabetes was reported in 155 patients (37.6%) of patients, 23.8% of EOPAC vs. 41.1% in AOPAC had diabetes at diagnosis but with no statistical difference (*p* = 0.82). One patient of EOPAC had a positive family history of pancreatic cancer in a first degree relative (1.2%) compared to 5 patients in the AOPAC group (1.5%), no statistical differences were noted regarding family history of pancreatic cancer or history of malignancies other than pancreatic cancer in first degree relatives between the two study groups (Table 1).

No significant differences regarding other risk factors as obesity, smoking and alcohol consumption. Despite significantly higher mean weight at diagnoses in EOPAC (66.2 kg vs. 62.1 kg in AOPAC), the mean body mass index at diagnosis (BMI) was not statistically different (23.6 vs. 23.2, *p* = 0.24).

### Presentation

The median duration of symptoms before diagnosis was significantly longer in EOPAC (2 months in EO; range 5–12 months) compared to AOPAC (1 month in average age; range 3–12 months), *p* = 0.03. Abdominal pain was the most common presenting symptom, followed by jaundice and weight loss in both groups as shown in Table 1. EOPAC patients tend to present with a good PS (83% with ECOG PS = 1 or less), while 34% of AOPAC present with a ECOS PS 2 or more (*p* = 0.001).

### Clinicopathological characteristics

EOPAC patients presented more with localized resectable (23.8%) and locally advanced tumors (28.5%) compared to AOPAC (14.3% with localized resectable disease, 17.6% with locally advanced disease). AOPAC presented more with metastatic disease (64% vs. 45.2%, *p* = 0.003).

No significant differences in tumor characteristics including tumor site, size, grade, and neuroendocrine differentiation (Table 2). However, EOPAC patients with tubular, papillary and acinar adenocarcinoma were significantly higher than AOPAC (*p* = 0.001). Germline or somatic molecular testing for BRCA1/2, PALB2, NTRK were not offered to any patient.

**Table 2:** Tumor stage and pathological characteristics in EOPAC compared to AOPAC

	EOPAC (*n* = 84)	AOPAC (*n* = 328)	*p*
**Stage at diagnosis.**			0.003
Localized (resectable).	20 (23.8%)	47 (14.4%)	
Locally advanced/borderline resectable.	24 (28.6%)	58 (17.7%)	
Metastatic	38(45.2%)	210 (64%)	
Missing.	2(2.4%)	13 (3.9%)	
**CA19-9 (Median-range**)	135.5 (0.3-145851)	248 (0.4-600000)	0.120
**Tumor site.**			0.803
Head	53 (63%)	208(63.4%)	
Body	12 (14.8%)	67 (20.4%)	
Tail	8 (9.3%)	35 (10.7%)	
Non specified.	11(13%)	18 (5.4%)	
**Tumour size (cm)**			
Median (range)	4.2 (1.4–13)	4.2 (0.7–19)	0.699
**Histopathological subtype.**			
Adenocarcinoma	58 (69%	280(85.3%)	
Others (tubular, papillary and acinar).	19 (22.6%)	26 (7.9%)	0.001
unknown	7 (8.3%)	22 (6.7%)	
**Grade.**			0.233
Well differentiated	5 (5.9%)	18(5.4%)	
Moderate differentiated	47 (55.9%)	188 (57.4%)	
Poorly differentiated	11 (13.1%)	57 (17.4%)	
Not reported	21(25%)	65(19.8%)	
**Neuroendocrine differentiation.**			0.269
Yes	4 (4.7%)	8 (2.4%)	
No	71 (84.6%)	292 (89%)	
Missing	9 (10.7%)	28 (8.5%)	

Liver was the most affected site by metastases in both groups (52.1% of EOPAC,55.9% OF AOPAC), followed by abdominal lymph nodes (4.2% of EOPAC, 3.5% of AOPAC) and peritoneal involvement (2.1% of EOPAC, 3.1% of AOPAC) with no statistical difference (*p* = 0.94).

### Treatment of patients with localized resectable and borderline resectable PAC

For patients with localized resectable tumors, 95% of EOPAC underwent upfront surgical resection compared to 89.3% of AOPAC (*p* = 0.17). Adjuvant chemotherapy was offered to 55% of EOPAC and 51% of AOPAC with a median number of 6 cycles of treatment in both groups with no statistical difference (Table 3). For patients with locally advanced /borderline resectable tumors, all patients in the EOPAC were offered neoadjuvant chemotherapy compared to 82.7% of AOPAC. Four patients (6.8%) of AOPAC group received concurrent chemoradiotherapy as a definitive treatment, 10.2% of AOPAC received best supportive care for being either medically unfit for surgery or presenting with poor PS (ECOG PS 3–4).

**Table 3:** Therapeutic interventions for pancreatic cancer according to age group and stage at diagnos

	EOPAC (*n* = 84)	AOPAC (*n* = 328)	*p*
**Treatment for patients presenting with localized resectable disease** (*n* = **67**).			
**Upfront surgery**			0.17
Yes	19 (95%)	41(89.3%)	
No	0 -	6 (10.7%)	
unknown	1 (5%)	0	
**Adjuvant chemotherapy.**			0.573
Yes.	11 (55%)	24 (51%)	
No.	4(20%)	7 (14.9%)	
Missing	5(25%)	16 (34.1%)	
**Number of cycles**			
Median (range)	6 (1–8)	6 (1–6)	0.9
***Treatment for patients with locally advanced and borderline resectable disease at diagnosis*** (***n*** = ***82***).			
**Preoperative treatment.**			0.257
Neoadjuvant chemotherapy.	24 (100%)	48 (82.7%)	
Pre-operative CCRTH	0	4 (6.8%)	
BSC	0	6 (10.2%)	
**Type of neoadjuvant chemotherapy**			0.57
Gemcitabine single agent.	11 (45.8%)	26 (54.1%)	
FOLFIRINOX	9 (37.5%)	12 (25%)	
Gemcitabine/cisplatin.	4(16.75)	7 (14.5%)	
unknown	-	3 (6.45%)	
**Conversion to resectability.**	4 (19%)	2 (5.3%)	0.112
**Treatment for patients with metastatic disease.**			
**First line systemic therapy for mets disease** (***n*** = **273**).			
Chemotherapy.	43 (84.3%)	183 (82.4%)	
BSC	1 (2%)	13 (5.8%)	0.001
Missing	7 (13.7%)	26 (11.7%)	
Median duration of treatment (in months).	3 (1–11)	3 (1–7)	0.278
**Types of chemotherapy used in first line treatment.**			
Gemcitabine.	5 (11.6%)	122(62.2%)	< 0.001
Gem/cisplatin	11(25.6%)	18(9.25)	
Gem/cap	2 (4.7%)	7 (3.6%)	
FOLFIRINOX	15 (34.9%)	14(7.1%)	
GEMOX	2(4.7%)	4(2%)	
BSC	1(2.35)	13(6.6%)	
**Second line systemic therapy for mets disease** (***n*** = **99**).			-----
Chemotherapy.	16 (66.6%)	55 (73.4%)	
BSC	1 (4.2%)	10 (13.3%)	
Missing	7 (29.2%)	10 (13.3%)	
**Third line systemic therapy for mets disease** (***n*** = **32**).			
Chemotherapy	3 (27.3%)	8 (38.1%)	----
BSC	7 (63.6%)	6 (28.6%)	
Missing	1 (9.1%)	7 (33.3%)	

Adjuvant chemotherapy with single agent gemcitabine was the most administered regimen in both groups after curative resection (45.5% of EOPAC, 58.3%), followed by Gem/cap (36.4% of EOPAC, 25% of AOPAC) and finally FOLFIRINOX (18.2% of EOPAC, 16.7% of AOPAC) with no statistical differences (*p* = 0.88). For patients with locally advanced/borderline resectable tumors, FOLFIRINOX was more administered as neoadjuvant therapy in EOPAC (37.5%) compared to 25% of AOPAC (*p* = 0.57). (Table 3). Pre-operative CCRTH was the treatment of choice for 4 patients of the AOPAC group (6.8%), but not offered to any EOPAC patient (*p* = 0.257).

### Treatment of patients with metastatic disease

For patients presenting with metastatic disease and patients who relapsed after treatment for localized disease, 5.8% of AOPAC received best supportive care as the treatment choice in first line compared to 2% of EOPAC (*p* = 0.001). Ninety-nine patients out of 273 who received first line treatment for metastatic disease (36.2%) were evaluated to receive second line chemotherapy, with best supportive care offered in 4.2% of EOPAC and 13.3% of AOPAC. Only 11 patients received third line systemic treatment (3 of EOPAC and 8 of AOPAC), number were small for testing for statistical significance. Gemcitabine single agent was the most administered chemotherapeutic regimen in AOPAC (62.2%), FOLFIRINOX was the most used in EOPAC (34.6%), followed by Gem/cisplatin (25.6%). FOLFIRINOX and gemcitabine-based combinations with platinum agents were significantly more administered in EOPAC (p = < 0.001). Non of the patients received genomic testing or received Olaparib.

### Disease free survival (DFS), Progression free survival (PFS) and overall survival (OS)

**Fig. 1:**
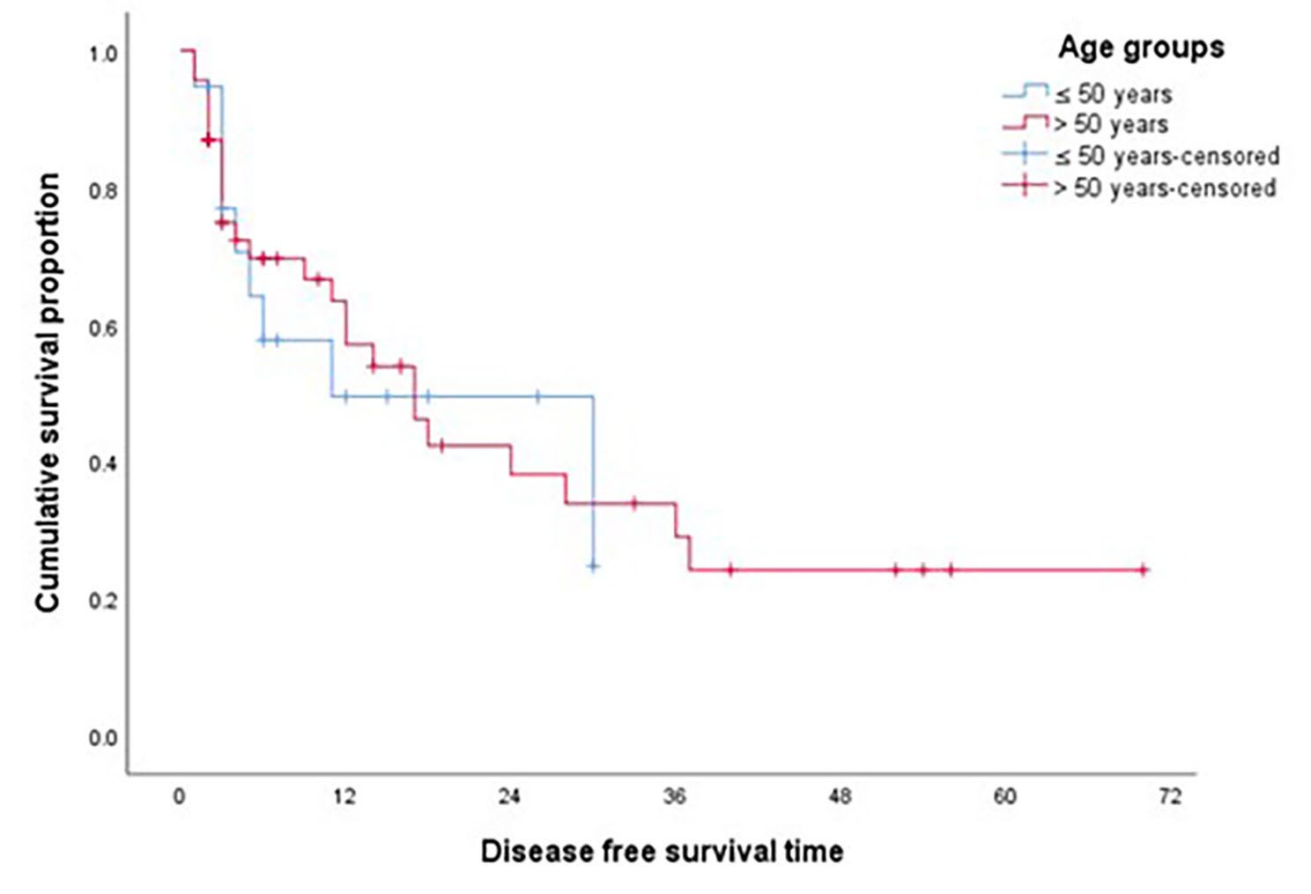
DFS after curative resection according to age groups

For the whole study group, the median DFS for patients who underwent surgical resection was 17 months. EOPAC patients had a shorter DFS of 11 months after curative resection compared to 17 months in average age group, but not statistically significant (*p* = 0.889) as shown in Fig. 1. Median PFS for patients receiving first line systemic treatment (PFS1) was similar between both groups (4 months in EOPAC,3 months in 3 AOPAC), also PFS for patients receiving second line treatment (PFS2) with median of 3 months for both EOPAC and AOPAC.

**Fig. 2:**
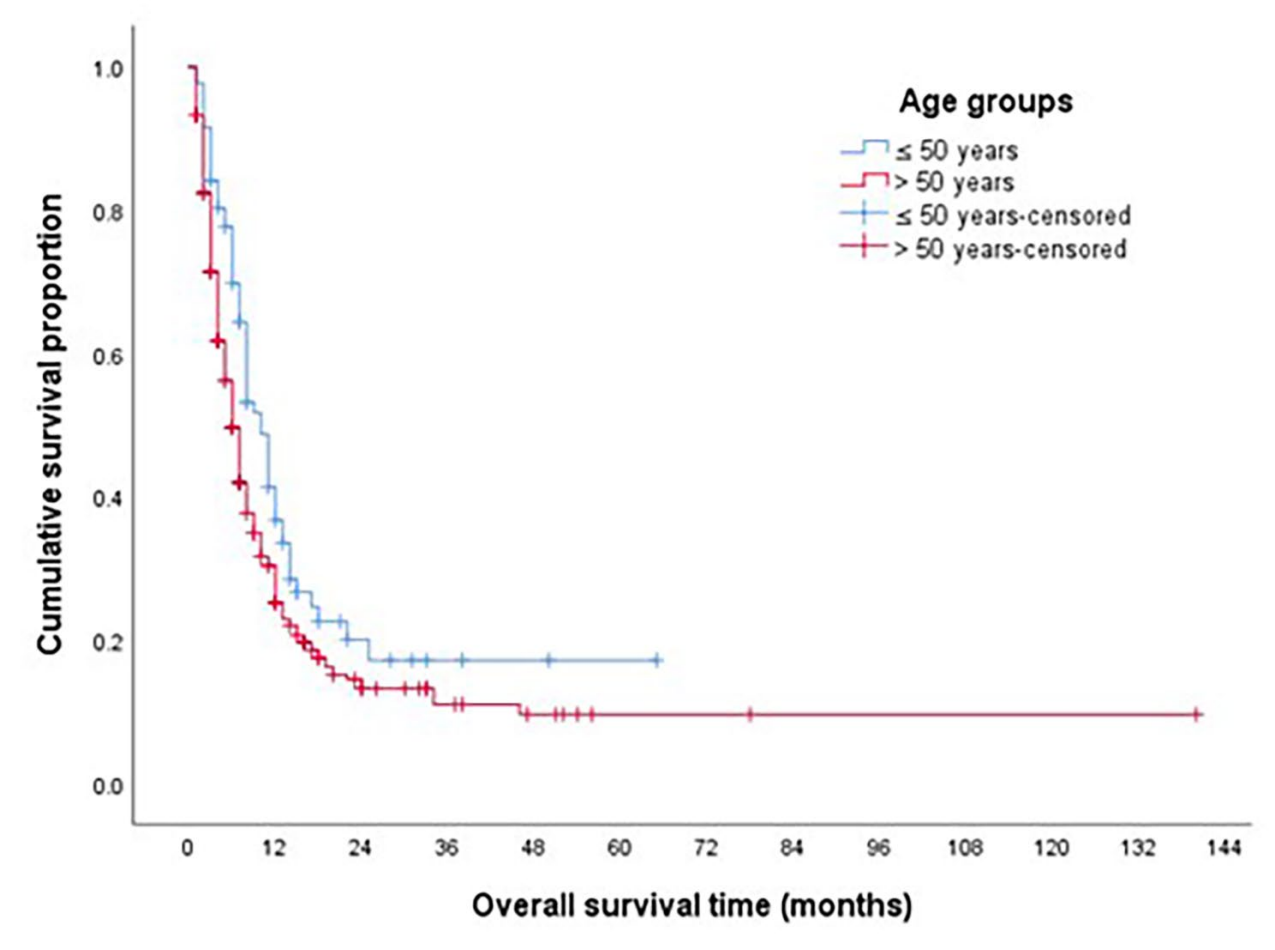
OS according to the age groups

**Fig. 3:**
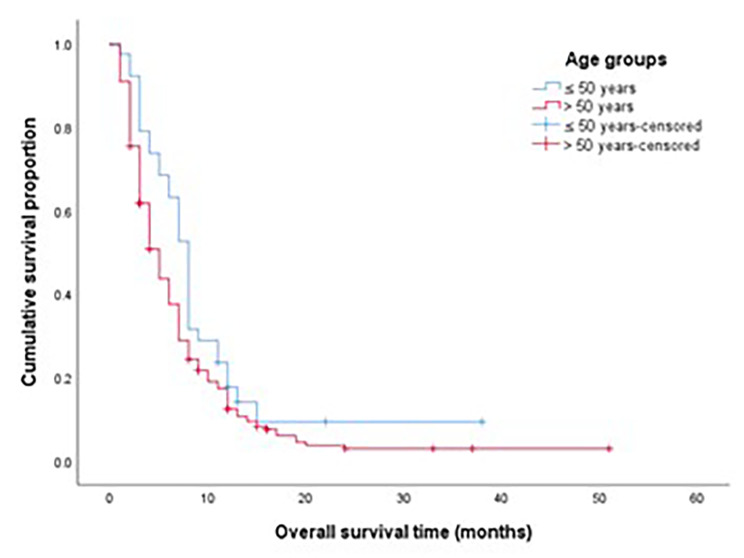
OS in patients presenting with localized disease according to age group

**Fig. 4:**
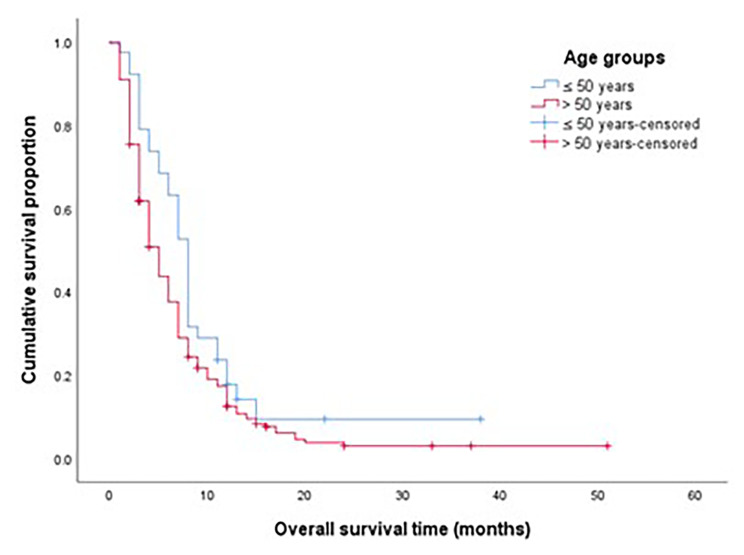
OS for patients presenting with metastatic disease according to age groups

The median OS for the whole study group was 7 months, the OS for EOPAC was significantly longer compared to AOPAC (10 months vs. 6 months, *p* = 0.013) as shown in Fig. 2. On multivariate analysis, the OS for patients presenting with localized disease was not different between both groups (Fig. 3), while OS was significantly longer in EOPAC group presenting with metastatic disease (8 months vs. 5 months, *p* = 0.39) as shown in Fig. 4.

## Discussion

Pancreatic adenocarcinoma ranks 12th in the most commonly diagnosed cancers worldwide [1]. The incidence and mortality related to pancreatic adenocarcinoma has doubled globally from 1990 to 2017, especially in women. The trends of increase in incidence and mortality varies geographically with the main increase reported in high sociodemographic index and developed countries [6, 18, 19]. There is a lack of data about the epidemiology of pancreatic cancer in Africa and the MENA region, with few publications covering the incidence and possible etiological factors [20]. We conducted this study to describe the clinical features and survival of EOPAC patients compared to AOPAC as well as possible etiological and risk factors. This was justified by early literature suggesting a potentially high proportion of PAC patients of young onset pancreatic cancer in Egypt [14] 43as well as possible different geographical variation in incidence [14–16]. 

The definition of early onset pancreatic cancer differs among studies, referring to ≤ 50 years old at diagnosis by most cohorts [8, 22, 23]. Other studies used the term (EOPAC) to describe patients ≤ 40 years [7]. In this study, we preferred to use ≤ 50 years as a cut-off point defining the EOPAC group. First because it is the most commonly used in literature and also because it was used in earlier studies on pancreatic cancer in Egypt [14]. 

EOPAC represents 20.3% of our study population, which is higher than that reported in literature (less than 10% of cases) [9]. However, our results are consistent with what was previously published from Egypt with more than one fourth of cases ≤ 50 years [14, 16]. It is not known if this is reflecting a true high incidence of EOPAC among Egyptians, or attributed to the inverted population pyramid in Egypt with the majority of population are of young age [24]. 

The prevalence of known risk factors for PAC including smoking, alcohol consumption, diabetes and obesity for PAC was similar in both study groups. Which is consistent with previous published data suggesting no significant differences in risk factors involved in development of PAC in EOPAC compared to AOPAC [10]. Males were more frequently affected in both groups (60.7% of EOPAC compared to 62.2% of AOPAC) but with no significant differences in gender distribution between both groups. This contradicts some studies that suggested a higher incidence of EOPAC among males compared to AOPAC [7, 23], other cohorts reported increasing incidence of EOPAC among females [6]. Hereditary cancer syndromes have been associated with development of PAC at a young age and a higher prevalence of germline mutations compared to average onset PAC patients [25, 26]. Criteria suggesting familial pancreatic cancer defined as having as at least two first-degree relatives (FDR) with pancreatic cancer without an identifiable syndrome or genetic mutation in the family was not identified in any patient in both EOPAC and AOPAC^41^,^42^. However, the numbers with reported family history of PAC were so small to draw conclusions (1 patient in EOPAC, 5 patients in AOPAC).

Patients with early onset pancreatic cancer tend to have symptoms for a longer duration compared to average onset and elder patients [27, 28]. This could be attributed to low level of clinical suspicious and awareness of physicians with alarming symptoms of cancer in early onset cancer patients, considering cancer a disease of elderly [29]. In concordance with published data, abdominal pain was the most common presenting symptoms followed by jaundice and weight loss with no significant differences between EOPAC and AOPAC [7, 8, 22, 23]. Also, EOPAC have a significantly better ECOG PS at diagnosis in our study as in other studies [23]. This delay in diagnosis was not reflected on the stage at diagnosis in the current study, as the rate of patients presenting with localized disease amenable for resection was significantly higher in EOPAC. Unlike other studies, AOPAC presented with metastatic disease were significantly higher in number compared to EOPAC. Most of the studies reported a more advanced stage at diagnosis in EOPAC when compared to AOPAC, with frequently higher incidence of metastatic disease in EOPAC [7, 9, 22, 23, 30]. 

In a review by Primavesi and collegues [9], summarizing studies on EOPAC. No significant differences were noted in clinicopathological features of EOPAC compared to AOPAC including tumor site (head, body, and tail), differentiation, lymphovascular and perineural invasion. Our findings are consistent with these data with no significant differences noted except the higher incidence of rare histopathological variants of adenocarcinoma in EOPAC. Also, no differences in median CA19-9 level at diagnosis or sites of metastases in patients with metastatic disease.

EOPAC patients tend to present with a better PS and less comorbidities compared to AOPAC [13]. This could explain the higher frequency of EOPAC patients submitted to radical surgery for resectable disease, receiving neoadjuvant and adjuvant chemotherapy with intensified regimens (FOLFIRINOX). Also, EOPAC patients received more aggressive therapies in first line in the form of FOLFIRINOX and Gemcitabine/cisplatin compared to AOPAC who predominantly received gemcitabine single agent for treatment of metastatic disease. Same patterns of care offered to EOPAC were reported in literature [23]. 

Despite the more aggressive treatment approaches for EOPAC with localized and locally advanced borderline disease, DFS in EOPAC patients submitted to surgery was worse. Our findings contradicts other trials that reported a longer DFS in EOPAC who underwent surgical resection of localized tumors especially in stage I-II disease [31, 32]. Same finding was reported by other trials [7, 9]. Trials explored the possibility of over-treatment for early onset tumors due to physician bias, on stage adjusted survival, no OS benefit was noted despite more aggressive treatment [33], it is not known if this applies to EOPAC as well.

A longer OS reported for EOPAC, which could be attributed to more intensified treatment received by EOPAC with treatment regimens that shown OS benefit in clinical trials as FOLFIRINOX [34] and Gem/cisplatin [35]. It is to be noted that the benefit seen with gem/cisplatin in clinical trials was mainly reported in patients harboring BRCA/PALP2 mutation [36], which is more predominant as a somatic as well as a germ line mutation in younger population [37]^40^. The prevalence of BRCA/PALP2 mutations is not known in our population so the long OS seen couldn’t be attributed to the presence of mutation. Germline and somatic testing for all patients eligible for systemic treatment is recommended by international guidelines [24, 38, 39]. But it was underutilized in our center due to economic barriers and lack of governmental re-imbursement for PARP inhibitors. It’s also not attributed to subsequent lines of treatment as there was no statistical difference in number of patients who crossed to second and third lines of treatment. Also, the number of patients who received third line treatment was so small that is not allowing for drawing conclusions.

Age adjusted incidence rates are required to estimate the real incidence of EOPAC in Egypt, as well as possible etiological and risk factors. The current study main limitation is the under-reporting of family history, risk factors and absence of genomic data.

## Conclusion

Data on the incidence and disease characteristics of pancreatic cancer in general and early onset pancreatic cancer are scarce in the MENA region. Patients with EOPAC have the same risk factors and etiological factors as AOPAC, most of cases are sporadic. The current study highlights the high proportion of EOPAC in Egyptian patients. Despite the similar clinic-pathological features, EOPAC patients tend to have a shorter DFS but longer OS.

## Data Availability

No datasets were generated or analysed during the current study.

## Abbreviations

AOPAC: Average onset pancreatic cancer
BRCA 1/2: Breast cancer associated gene
DFS: Disease free survival
EOPAC: Early onset pancreatic cancer
MENA: Middle east and north Africa region
OS: Overall survival
